# Differential regulation of TNF-α and IL-1β production from endotoxin stimulated human monocytes by phosphodiesterase inhibitors

**DOI:** 10.1155/S0962935192000620

**Published:** 1992

**Authors:** K. L. Molnar-Kimber, L. Yonno, R. J. Heaslip, B. M. Weichman

**Affiliations:** Inflammation/Bone Metabolism Division Wyeth-Ayerst Research CN 8000 Princeton NJ 08543-8000 USA

## Abstract

The effect of selective PDE-I (vinpocetine), PDE-III (milrinone, CI-930), PDE-IV (rolipram, nitroquazone), and PDE-V (zaprinast) isozyme inhibitors on TNF-α and IL-1β production from LPS stimulated human monocytes was investigated. The PDE-IV inhibitors caused a concentration dependent inhibition of TNF-α production, but only partially inhibited IL-1β at high concentrations. High concentrations of the PDE-III inhibitors weakly inhibited TNF-α, but had no effect on IL-1β production. PDE-V inhibition was associated with an augmentation of cytokine secretion. Studies with combinations of PDE isozyme inhibitors indicated that PDE-III and PDE-V inhibitors modulate rolipram's suppression of TNF production in an additive manner. These data confirm that TNF-α and IL-1β production from LPS stimulated human monocytes are differentially regulated, and suggest that PDE-IV inhibitors have the potential to suppress TNF levels in man.


Mediators of Inflammation 1, 411–417 (1992)

### Differential regulation of TNF-α and IL-1β production from endotoxin stimulated human monocytes by phosphodiesterase inhibitors

K. L. Molnar-Kimber,[CA] L. Yonno, R. J. Heaslip and B. M. Weichman

Inflammation/Bone Metabolism Division, Wyeth-Ayerst Research, CN 8000, Princeton, NJ 08543-8000, USA

[CA] Corresponding Author

**THE effect of selective PDE-I (vinpocetine), PDE-III (milrinone, CI-930), PDE-IV (rolipram, nitroquazone), and PDE-V (zaprinast) isozyme inhibitors on TNF-α and IL-1β production from LPS stimulated human monocytes was investigated. The PDE-IV inhibitors caused a concentration dependent inhibition of TNF-α production, but only partially inhibited IL-1β at high concentrations. High concentrations of the PDE-III inhibitors weakly inhibited TNF-α, but had no effect on IL-1β production. PDE-V inhibition was associated with an augmentation of cytokine secretion. Studies with combinations of PDE isozyme inhibitors indicated that PDE-III and PDE-V inhibitors modulate rolipram's suppression of TNF-α production in an additive manner. These data confirm that TNF-α and IL-1β production from LPS stimulated human monocytes are differentially regulated, and suggest that PDE-IV inhibitors have the potential to suppress TNF-α levels in man.**

**Key words:** cAMP, Cytokine, Endotoxin, Human monocytes, Interleukin 1β, Phosphodiesterase inhibitors, Tumour necrosis factor-α.

### Introduction

Activation of monocytes results in the secretion of numerous cytokines, including Il-1 and tumour necrosis factor α (TNF-α), which play key roles in regulation of the immune system.[1,2] However, unregulated overproduction of these cytokines has been cited as a major factor in the development of septic shock, rheumatoid arthritis and inflammatory lesions.[1,2] Thus, the production of these cytokines must be tightly controlled by numerous mechanisms. Macrophage activation induces a rise in the rate of transcription for IL-1 and TNF-α mRNA and a concurrent increase in the stability[1,3] and translational efficiency of TNF-α mRNA.[1] The precursors of the cytokines are enzymatically cleaved, and TNF-α and IL-1β are released into the interstitial spaces or blood.[1,2] Typically, monocyte activation is associated with a 200–3000 fold and 150–1000 fold increase in TNF-α and IL-1β production, respectively. Although endotoxin induces monocytes to secrete both IL-1β and TNF-α, the production of these cytokines has been shown to be differentially regulated.[3–5] For example, IL-1, but not TNF-α, production by endotoxin induced monocytes can be selectively decreased by a calmodulin dependent kinase inhibitor, H7.[5] Cellular activation is often accompanied by changes in the intracellular levels of cyclic nucleotides, and phosphodiesterases, by metabolizing the intracellular cyclic nucleotides, play a major role in modulating their intracellular concentrations. The nonspecific phosphodiesterase (PDE) inhibitors, theophylline, 3-isobutyl-1-methylxanthine (IBMX), and pentoxifylline, selectively decrease TNF-α mRNA steady state levels and TNF-α production from endotoxin-stimulated murine and human monocytes.[6–8] However, the effect of agents which augment intracellular levels of cAMP on IL-1β production from LPS induced macrophages varies depending on the species, stimulus, and culture conditions;[2] results ranging from stimulation[9] to no effect[8] to inhibition[10–12] have been reported.

There are five distinct families of phosphodiesterase (PDE) isoenzymes which differ in their cyclic nucleotide substrate specificities and tissue distribution.[13,14] Selective inhibitors for PDE-I, PDE-III, PDE-IV and PDE-V have been identified, although the selective inhibitors may display some inhibitory effect on other PDE isoenzymes at higher concentrations.[15] PDE-IV has been isolated from human monocytes,[16] and PDE activity was also detected in peaks eluting in buffers for PDE-I and PDE-V isolation from murine macrophages.

This study was undertaken to investigate the role of cAMP, cGMP and PDE isoenzymes in regulation of monocyte cytokine production. The effect of selective inhibitors of the PDE-I, PDE-III, PDE-IV and PDE-V isotypes on intracellular and secreted TNF-α and IL-1β production from endotoxin stimulated human monocytes was analysed. Interactions between the regulation exerted by PDE isoenzymes were investigated in

© 1992 Rapid Communications of Oxford Ltd combination studies with PDE-I, PDE-III, or PDE-V inhibitors and PDE-IV inhibitor.

### Materials and Methods

*Reagents:* CI-930 (Parke Davis Pharmaceutical Research, Ann Arbor, MI), milrinone, (Sterling Winthrop Research Institute, Rennsalear, NY), rolipram (Schering AG, Berlin, Germany), nitraquazone (Troponwerke, Nordrhein-Westfalen, Germany), and zaprinast (May and Baker, Essex, UK) were generously provided by their respective manufacturers. Vinpocetine was made by Wyeth-Ayerst Research, Princeton, NJ. Dexamethasone was purchased from Sigma (St Louis, MO) and stored at 4°C. Each drug was dissolved in 100% ethanol or DMSO to yield a final concentration of 10 mM. The stocks were diluted just before use with culture media, consisting of RPMI 1640 (Gibco, Gaithersburg, MD) supplemented with 5% foetal calf serum (FCS; Hyclone, Logan, UT), 25 mM HEPES, 2 mM L-glutamine, 100 $\mu$g Na-pyruvate, 100 U/ml penicillin, and 100 $\mu$g/ml streptomycin (RPMI 1640-5% FCS). Three different types of lipopolysaccharide (LPS), 0111:B4, 055:B5, and 026:B6 were purchased from List Biologicals (Campbell, CA), Sigma (St Louis, MO) and Difco (Detroit, MI), respectively for optimization of the assay. LPS was dissolved in $Ca^{2+}$, $Mg^{2+}$-free Dulbecco's phosphate buffer saline (PBS) (Gibco) to produce a stock containing 5 mg/ml, diluted to 1 mg/ml in PBS, aliquoted and then stored frozen at −20°C. LPS was thawed and diluted with RPMI 1640-5% FCS as needed.

*Human monocyte assay:* Human mononuclear cells were isolated by Ficoll hypaque density centrifugation (specific gravity 1.077) from Leukapaks obtained from healthy Caucasian males. The buffy coat layer was washed three times in RPMI 1640 and enumerated. Human mononuclear cells were plated into 24-well plates ($10^6$ cells in 1 ml RPMI 1640-5% FCS/well) or flat-bottom microtitre plates ($3.76 \times 10^5$ cells in 100 $\mu$l/ well) and incubated at 37°C in 5% $CO_2$ in a humidified atmosphere for 45 min. The wells were rinsed three times to remove non-adherent cells. The quantity of baseline and LPS stimulated cytokine production was measured from cells incubated in the absence or presence of 0.1 ng LPS (0111:B4 subtype) per ml, respectively, for 16–24 h at 37°C in 5% $CO_2$ in a humidified atmosphere. To assess the effect of drugs, varying concentrations of compounds or vehicle were added to the cells just prior to their incubation with 100 pg LPS/ml in RPMI 1640-5% FCS as above. All tests were performed in duplicate. To measure the concentration of secreted and cell associated IL-1$\beta$ and TNF-$\alpha$, supernatants were harvested. To assess cell associated cytokine levels, RPMI 1640 (0.3 ml) was added to the cells. Samples were frozen at −80°C and stored. Cell lysates were prepared by thawing plates, scraping cells from the well, adding 0.2 ml RPMI 1640, scraping remaining cells from the wells, and pooling samples followed by two additional freeze–thaw cycles.[17] The cell lysate was diluted two-fold with RPMI 1640 containing 10% FCS to yield a final volume of 1 ml. It should be noted that the effect of nitraquazone on monokine secretion was assessed in a set of experiments ($n = 3$) in which samples were not processed for analysis of cell associated monokine levels.

The viability of the LPS stimulated cells in the presence or absence of a test compound was assessed in the microtitre plates following a 16–24 h incubation at 37°C in 5% $CO_2$ using the previously described assay based on the metabolic reduction of 3-(4,5-dimethyl-thiazol-2yl)-2,5 diphenyl tetrazolium bromide (MTT).[18] None of the PDE inhibitors tested at the same concentrations decreased the viability of the monocytes.

*IL-1$\beta$ and TNF-$\alpha$ assays:* The concentrations of IL-1$\beta$ and TNF-$\alpha$ in the supernatants and cell lysates were measured in duplicate using ELISA kits (Cistron, Pine Brook, NJ). The standards, supernatants and cell lysates were diluted with RPMI 1640-5% FCS just before use. After the addition of the samples and standards to the appropriate wells, the plates were incubated at 37°C overnight in a humidified atmosphere containing 5% $CO_2$. The plates were then processed according to the manufacturer's instructions. However, the analysis was performed as listed below. The standard curve was fit to the following equation:

$$OD = C + (D - C)/ [1 + \mathrm{Exp}\,(-(A + B^* \,\mathrm{Lgt}\,(\text{drug conc})))]$$

Where OD was set to the optical density, C the estimated minimum, D the estimated maximum and A and B are derived as parameters of intercept and slope, respectively.

The concentration of cytokine was calculated by inverse prediction. Percent inhibition was calculated as follows:

$$\%\ \text{Inhibition} = \left(1 - \frac{[\text{test}] - \text{baseline}}{\text{stimulated} - \text{baseline}}\right) \times 100$$

The $IC_{50}$ value, defined as 50% inhibition of the stimulated control response was determined by inverse prediction of the following equation:

$$\text{Concentration} = C + D$$

Where D equals the cytokine concentration in the stimulated control less that of the baseline control.

*Statistics:* To determine whether the individual PDE inhibitors significantly blocked TNF-α and IL-1β production and/or secretion, an analysis of variance for a randomized block design (using each donor as a block) was performed.[19] This included all treatments where a single PDE inhibitor was given. Similarly, to assess whether the PDE-I, PDE-III and PDE-V inhibitors could modulate the inhibition of TNF-α by rolipram alone, a similar analysis was performed. A separate analysis was performed for each combination, and all treatment groups including either drug alone or in combination were included. A *t*-statistic taking into account the within- and between-donor variation was used to test whether the percent inhibition observed was significantly different from zero. Degrees of freedom were approximated by Satterwaite's formula.[20] Interaction between drugs was tested by estimating the expected inhibition for the combination based on the inhibition for the individual PDE inhibitors and testing whether this was significantly different from the observed inhibition for the combination. Pairwise comparisons using the least significant difference method were performed to test whether the combination was better than either drug alone.

### Results

Wasik and Beller[21] have observed that the inhibitory effect of compounds on cytokine production may be minimized by overstimulating the monocytes. As an initial step, the quantity of endotoxin was titrated to determine the concentration required to stimulate near maximal release of IL-1β and TNF-α from human monocytes. As shown in Fig. 1, 0.1 ng/ml of lipopolysaccharide (LPS) induced near maximal release of both IL-1β and TNF-α, regardless of the LPS subtype. Therefore, 0.1 ng/ml LPS (subtype 0111:BB4) was used to assess the effect of drugs on cytokine release.

The quantity of IL-1β and TNF-α secreted by LPS stimulated monocytes increased by 100 to 1000-fold over the baseline level of 4–13 pg/ml and 5–23 pg/ml, respectively (data not shown). The cell associated TNF-α was less than 10% of the total produced, whereas the cell associated IL-1β ranged from 33.8% to 61.8% of the total produced. The level of TNF-α and IL-1β elicited by LPS stimulation of human monocytes varied from donor to donor, in agreement with prior reports.[22,23]

*Effect of PDE inhibitors on monokine release:* TNF-α secretion was significantly suppressed by the PDE-IV inhibitors, rolipram and nitraquazone, in a concentration dependent manner (Fig. 2A), with maximal inhibition of approximately 80% noted at 10 μM. The $IC_{50}$ values of rolipram and ni-

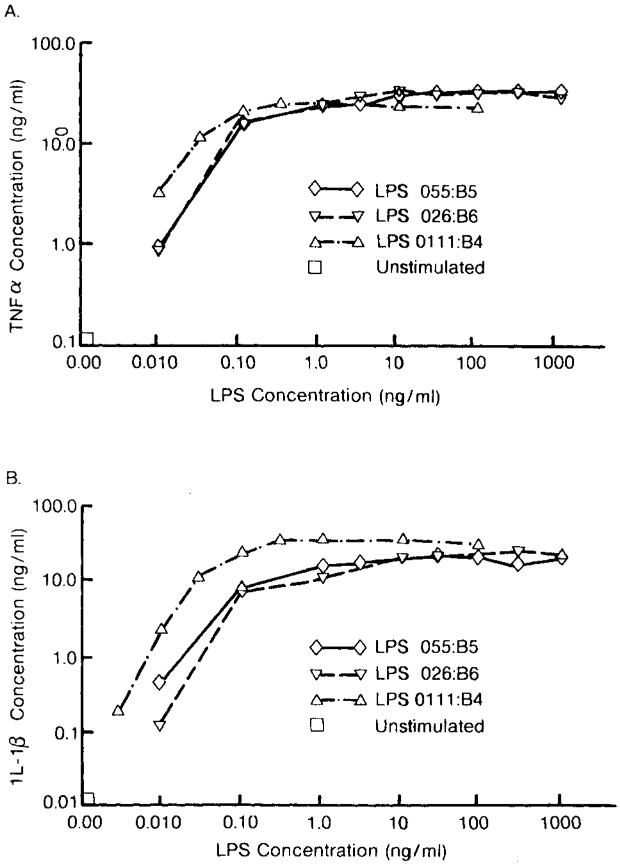

FIG. 1. Concentration response curve of lipopolysaccharide on cytokine production from human monocytes isolated from one donor. Similar response curves were observed with other donors. Effect of various concentrations of LPS on TNF-α secretion (A) and on IL-1β secretion (B) from stimulated human monocytes.

traquazone were 0.38 μM and 0.23 μM, respectively. The PDE-III inhibitors, CI 930 and milrinone, at 10 μM only suppressed TNF-α with a maximal inhibition of 24% and 13%, respectively. Although the PDE-I inhibitor, vinpocetine, did not affect TNF-α production, the PDE-V inhibitor, zaprinast, at 10 μM modestly but significantly augmented the TNF-α secretion. In agreement with previous reports,[23] dexamethasone exhibited an $IC_{50}$ of 0.01 μM for these same donors, with 90% inhibition of TNF-α noted at the highest concentrations used.

Rolipram at 10 μM and nitraquazone at 1 and 10 μM suppressed IL-1β secretion from LPS stimulated human monocytes ($n = 13$ and $n = 3$, respectively), although there was much variability in the sensitivity of the response to these drugs (Fig. 2B). For example, IL-1β inhibition at 10 μM rolipram or nitraquazone ranged from −43% to 53% and 14% to 57%, respectively. Vinpocetine, CI 930 and milrinone did not have a significant effect on IL-1β secretion. In contrast, zaprinast, a PDE-V inhibitor, significantly augmented the release of IL-1β from LPS stimulated human monocytes.

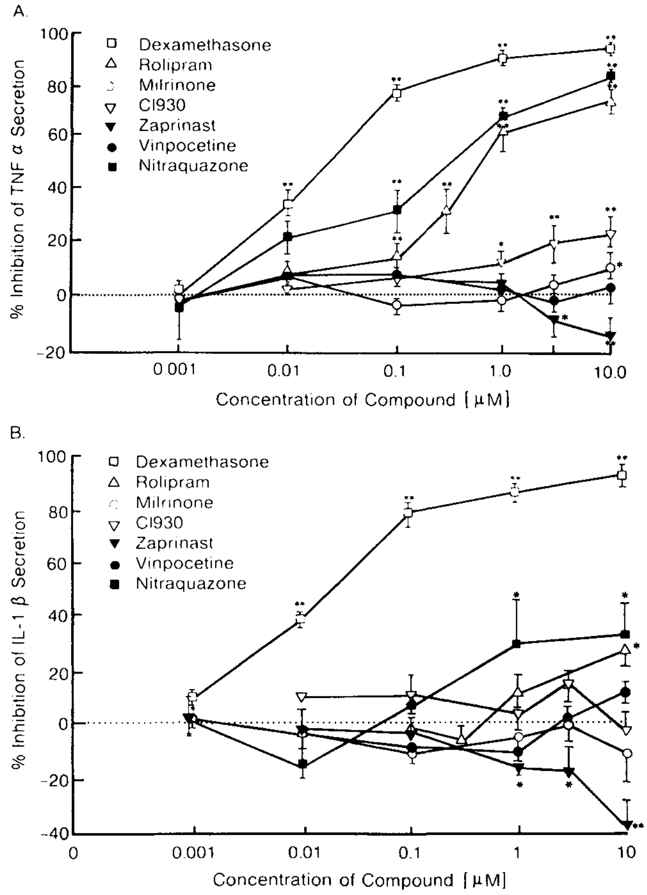

FIG. 2. The effect of dexamethasone and PDE inhibitors on TNF-α or IL-1β secretion from LPS induced human monocytes. The mean effect ± standard error of these drugs on TNF-α (A) and on IL-1β (B) secretion ($n \geq 3$) is plotted. Values were significantly different from the control at the $p = 0.05$ (*) or $p = 0.01$ (**) levels.

Zaprinast was ten times more potent in augmenting IL-1β secretion than TNF-α secretion. Dexamethasone suppressed IL-1β production ($IC_{50} = 0.02$ μM) as expected[8,23] (Fig. 3B).

The PDE inhibitors tested at the above concentrations did not decrease the viability of the cells as determined by the MTT assay. Therefore, the effect on cytokine production was not due to cytotoxicity.

*Combination studies:* In order to determine whether the PDE-I, PDE-III, PDE-IV or PDE-V inhibitors exerted their effects on TNF-α in an additive, synergistic or antagonistic manner, the effect of combinations of submaximal levels of rolipram (0.1 and 0.3 μM) with 3 and 10 μM concentrations of vinpocetine, CI 930, milrinone, and zaprinast was evaluated. As shown in Fig. 3, rolipram's inhibition of TNF-α release was increased by the presence of CI 930 (3 μM and 10 μM) and milrinone (10 μM) in an additive manner. Vinpocetine had no effect on rolipram's suppression of TNF-α. Zaprinast (10 μM) alone augmented TNF-α and in combination with rolipram decreased rolipram's inhibition of TNF-α secretion in an additive manner.

*Cell associated cytokines:* Cell associated cytokines were quantitated in order to probe the effect of the selective PDE inhibitors on the intracellular cytokine concentrations. Similar to their effects on secretion of TNF-α, both CI 930 and rolipram also decreased the level of TNF-α which was cell associated (Fig. 4A). Although zaprinast signi-

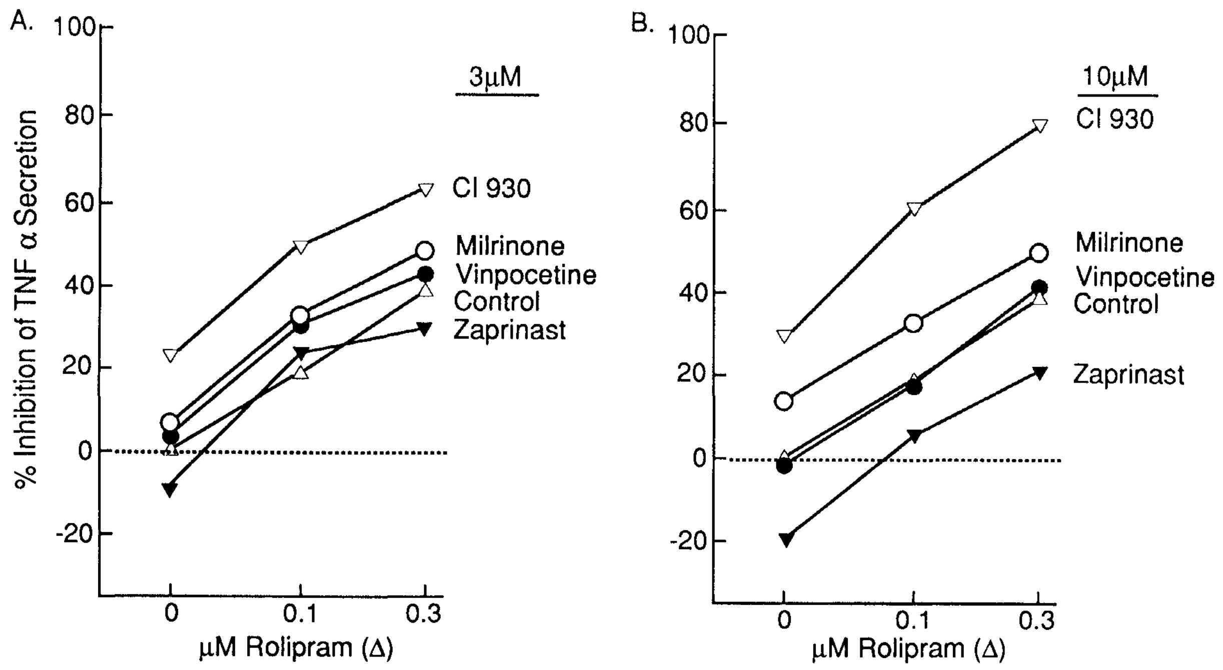

FIG. 3. The effect of PDE-I, III, and V inhibitors on rolipram's suppressive effect on TNF-α secretion. TNF-α in the absence (0 μM rolipram) or presence of (0.1 or 0.3 μM rolipram) the PDE-IV inhibitor is depicted by the curve labelled 'control' (△). The effect of other PDE isozyme inhibitors assessed at concentrations of 3 μM (A) or 10 μM (B) on the effect of rolipram's inhibition of TNF-α secretion is plotted. Vinpocetine (●), CI 930 (▽), milrinone (○), or zaprinast (▼) were the PDE isozyme inhibitors employed.

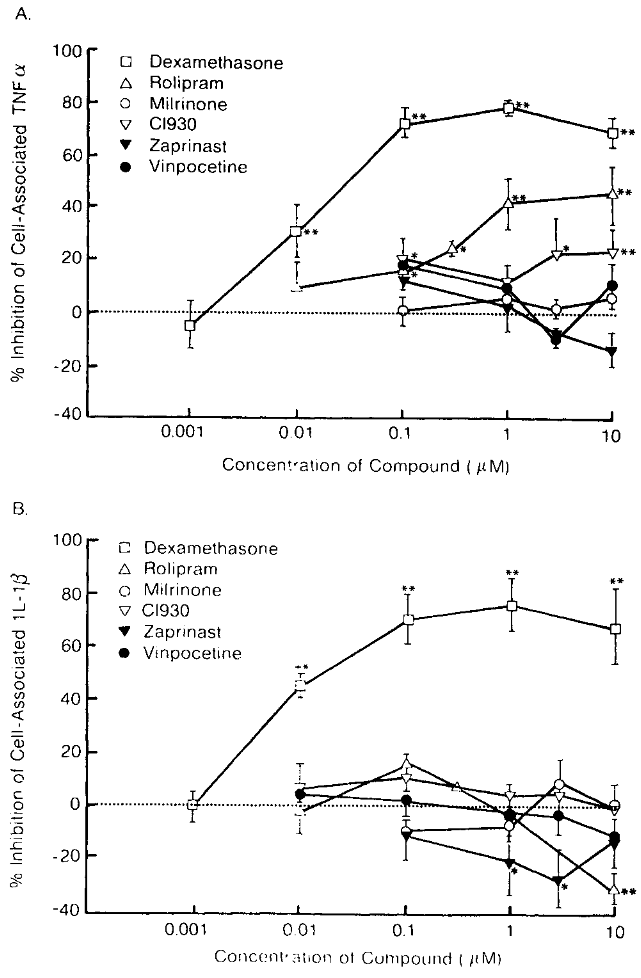

FIG. 4. The effect of dexamethasone and PDE inhibitors on production of cell associated TNF-α or IL-1β from LPS induced human monocytes. The mean effect ± standard error of these drugs on cell associated TNF-α (A) and IL-1β (B) ($n \geq 2$). Values were significantly different from the control at the $p = 0.05$ (*) or $p = 0.01$ (**) levels.

ficantly augmented secreted TNF-α production ($p = 0.004$), the apparent increase in cell associated TNF-α by zaprinast was not significant ($p = 0.2$).

Similar to zaprinast's effects on IL-1β secretion, the level of cell associated IL-1β was significantly augmented by zaprinast (Fig. 4B). Although vinpocetine, CI 930, and milrinone did not affect the intracellular levels of IL-1β, 10 μM rolipram significantly increased the concentration of cell associated IL-1β. This increase in cell associated IL-1β occurred at rolipram concentrations similar to those which inhibited IL-1β secretion. Dexamethasone significantly suppressed cell associated TNF-α and IL-1β ($IC_{50} = 0.02$ and 0.01 μM, respectively).

### Discussion

Agents which regulate intracellular cyclic nucleotide levels such as dibutryl cAMP (dbcAMP), prostaglandin $E_2$ ($PGE_2$) and phosphodiesterase inhibitors can modulate macrophage functions. Superoxide generation from guinea pig peritoneal macrophages was inhibited by rolipram and Ro-20-1724, two PDE-IV inhibitors, but not by SKF 94120, a PDE-III inhibitor.[24] Human alveolar macrophage activation was modestly suppressed by forskolin; Ro-20-1724 had no effect.[25] Agents which elevate cAMP levels, such as dbcAMP, isoproterenol, and nonselective PDE inhibitors (e.g. pentoxifylline, IBMX, and theophylline), suppressed the production of TNF-α from LPS induced monocytes and macrophages.[6–8,11] The present studies showed that selective PDE isoenzyme inhibitors can differentially effect the production of TNF-α and IL-1β from LPS induced human monocytes. Rolipram and nitraquazone, selective inhibitors of PDE-IV, suppressed production of secreted TNF-α with a maximal inhibition of approximately 80% (Fig. 3A). CI 930 and milrinone at concentrations of 3 and 10 μM modestly depressed TNF-α secretion. Although CI 930 and milrinone can inhibit PDE-IV enzymatic activity at concentrations greater than 100 μM and 10 μM, respectively,[15,26] the inhibition of TNF-α production by CI 930 occurred at lower concentrations. Thus, the effect of at least CI 930 is most likely caused by suppression of inherent PDE-III activity in the monocytes. The observation that combinations of these PDE-III inhibitors and rolipram resulted in additive suppression of TNF-α production are consistent with the expectation that these agents mediate their effect via the same mechanism (elevation of cAMP levels).

In contrast, zaprinast, a PDE-V inhibitor that is expected to regulate cGMP levels, modestly but significantly increased secreted TNF-α at 10 μM, in agreement with reports of other agents which elevate intracellular cGMP levels (sodium nitroprusside and exogenous cGMP) and which stimulate TNF-α production.[6] The observation that the combination of zaprinast with rolipram decreased the TNF-α inhibition observed with rolipram alone in an additive manner is consistent with opposing roles for PDE-V/cGMP and PDE-IV/cAMP in TNF-α secretion by endotoxin stimulated monocytes.

TNF-α production from activated macrophages is increased 100 to 5000-fold by a combination of elevated transcription rate, increased mRNA stability and improved translational efficiency.[1] Since cell associated as well as secreted TNF-α levels were inhibited by rolipram and CI 930, these drugs probably do not inhibit secretion by blocking precursor processing. The nonspecific PDE inhibitors, pentoxifylline and IBMX, have been shown to reduce steady state levels of mRNA and suppress TNF-α production by LPS stimulated monocytes and macrophages.[8] Elevation of intracellular cAMP levels in LPS stimulated murine macrophages by dbcAMP is also reported to suppress TNF-α production, lower the transcription rate and decrease steady state levels of mRNA.[8,11] Thus, one likely mechanism by which PDE-III and PDE-IV inhibitors may regulate TNF-α is by decreasing the steady state level of TNF-α mRNA in the cell.

The effect of cAMP modulating agents on IL-1β production from LPS activated monocytes varies with the culture conditions, type of stimulus and species.[2] Kassis *et al.*[9] observed that agents which elevate cAMP stimulate IL-1β production. Endres *et al.*[8] have observed no effect on total IL-1β protein or mRNA levels in LPS stimulated monocytes or macrophages in the presence of agents which elevated cAMP levels including nonspecific PDE inhibitors. In contrast, Brandwein,[27] Hurme,[12] and Knudsen and colleagues[10] reported that concurrent treatment of LPS induced macrophages with cAMP elevating agents result in a modest decrease in secretion of IL-1β. No effect on IL-1β mRNA steady state levels is observed with treatment with agents which elevate cAMP levels.[10–12] In the present studies, rolipram and nitraquazone at high concentrations did significantly decrease IL-1β secretion, in agreement with the studies of Knudsen, Hurme and Brandwein.[10,12,27] Since rolipram increased the quantity of cell associated IL-1β (nitraquazone was not tested), while it decreased secreted IL-1β, this data supports the interesting suggestion that PDE-IV may play a role in modulating IL-1β release from the cell.[10] This action by rolipram may help to account for some of the differences between previous reports which assessed the effects on secreted *vs.* total IL-1β production. Secondly, variability of sensitivity of IL-1β production to PDE-IV inhibitors between donor monocyte preparations may also account for some differences.

Disparate results are observed with cGMP elevating agents on IL-1β secretion where SIN-1 treatment inhibits IL-1β,[8] dibutryl cGMP has no effect[12] and in the present study, zaprinast increased IL-1β secretion. These disparities may have arisen due to different levels of stimulation and/or culture conditions. However, under the same conditions, the observations that PDE-IV and PDE-V inhibitors exerted opposite effects on IL-1β and TNF-α secretion raise the possibility that cAMP and cGMP may affect endotoxin induced cytokine secretion in an opposing manner.

The suppressive effect of selective PDE-IV inhibitors on TNF-α suggests that these and similar compounds may be useful in the prophylactic and/or therapeutic treatment of pathologies involving overproduction of TNF-α. Since anti-TNF-α reagents have been reported to decrease symptoms in animal models of septic shock, experimental allergic encephalitis, and collagen induced arthritis, PDE-IV inhibitors may have a therapeutic benefit in diseases such as arthritis, septic shock, and multiple sclerosis. PDE-IV inhibitors have been suggested also to be of potential benefit in the treatment of asthma, based upon their significant bronchodilatory and anti-inflammatory activities.[14,28] Although the role of TNF-α in asthma has not been clearly established, a recent report suggested that increases in bronchoalveolar lavage TNF-α levels are associated with exacerbations of asthma.[29] Furthermore, TNF-α was reported to stimulate eosinophil oxidant production and toxicity towards human endothelium.[30] Thus, it may also be suggested that the anti-TNF-α activities of PDE-IV inhibitors will contribute to their anti-asthma profiles. Further experiments will be needed to explore this possibility.

### References

1. Beutler B, Cerami A. The biology of cachetin/TNF-α primary mediator of the host response. *Ann Rev Immunol* 1989; **7**: 625–655.
2. Dinarello C. Interleukin 1 and interleukin 1 antagonism. *Blood* 1991; **77**: 1627–1652.
3. Fenton MJ. Review: Transcriptional and post-transcriptional regulation of interleukin 1 gene expression. *Int J Immunopharmac* 1992; **14**: 401–411.
4. Burchett SK, Weaver WM, Westall JA, Larsen A, Kronheim S, Wilson CB. Regulation of tumor necrosis factor/cachectin and IL-1 secretion in human mononuclear phagocytes. *J Immunol* 1988; **140**: 3473–3481.
5. Kovacs EJ, Radzioch D, Young HA, Varesio L. Differential inhibition of IL-1 and TNF-α mRNA expression by agents which block second messenger pathways in murine macrophages. *J Immunol* 1988; **141**: 3101–3105.
6. Renz H, Gong JH, Schmidt A, Nain M, Gemsa D. Release of tumor necrosis factor α from macrophages: enhancement and suppression are dose dependently regulated by prostaglandin $E_2$ and cyclic nucleotides. *J Immunol* 1988; **141**: 2388–2393.
7. Strieter RM, Remick DG, Ward PA, Spengler JP, Larrick J, Kunkel SL. Cellular and molecular regulation of tumor necrosis factor alpha production by pentoxifylline. *Biochem Biophys Res Commun* 1988; **155**: 1230–1236.
8. Endres S, Fuelle HJ, Sinha B, *et al.* Cyclic nucleotides differentially regulate the synthesis of tumor necrosis factor alpha and interleukin 1 beta by human mononuclear cells. *Immunology* 1991; **72**: 56–60.
9. Kaissis S, Lee JC, Hanna N. Effects of prostaglandins and cAMP levels on monocyte IL-1 production. *Agents and Actions* 1989; **27**: 274–276.
10. Knudsen PJ, Dinarello CA, Strom TB. Prostaglandins posttranscriptionally inhibit monocyte expression of interleukin 1 activity by increasing intracellular cyclic adenosine monophosphate. *J Immunol* 1986; **137**: 3189–3194.
11. Tannenbaum CS, Hamilton TA. Lipopolysaccharide induced gene expression in murine peritoneal macrophages is selectively suppressed by agents that elevate intracellular cAMP. *J Immunol* 1989; **142**: 1274–1280.
12. Hurme M. Modulation of interleukin-1β production by cyclic AMP in human monocytes. *FEBS Letters* 1990; **263**: 35–37.
13. Nicholson CD, Challiss RAJ, Shahid M. Differential modulation of tissue function and therapeutic potential of selective inhibitors of cyclic nucleotide phosphodiesterase isoenzymes. *Trends Pharmacol Sci* 1991; **12**: 19–27.
14. Giembycz MA. Commentary: Could isoenzyme-selective phosphodiesterase inhibitors render bronchodilator therapy redundant in the treatment of bronchial asthma? *Biochem Pharmacol* 1992; **43**: 2041–2051.
15. Beavo JA, Reifsnyder DH. Primary sequence of cyclic nucleotide phosphodiesterase isoenzymes and the design of selective inhibitors. *Trends Pharmacol Sci* 1990; **11**: 150–155.
16. Livi GP, Kmetz P, McHale MM, *et al.* Cloning and expression of cDNA for a human low-Km rolipram sensitive cyclic AMP phosphodiesterase. *Mol Cell Biol* 1990; **10**: 2678–2686.
17. Endres S, Ghorbani R, Lonnemann G, Van der Meer JWM, Dinarello CA. Measurement of immunoreactive interleukin 1β from human mononuclear cells: optimization of recover, intersubject consistency, and comparison with interleukin 1β and tumor necrosis factor. *Clin Immunol Immunopath* 1988; **49**: 428–438.
18. Mosmann TJ. Rapid colorimetric assay for cellular growth and survival: application to proliferation and cytotoxicity assays. *Immunol Meth* 1983; **65**: 55–63.
19. Finney DJ. *Statistical Method in Biological Assay*. Charles Griffin & Co., 1978.

20. Satterwaite FE. *Biometrics Bull* 1946; **2**: 110.
21. Wasik MA, Beller DI. Induction of macrophage membrane interleukin 1 expression by T-cell dependent and T-cell independent pathways is inhibited by cyclosporin A. *Clin Imm Immunopath* 1989; **52**: 331–340.
22. Endres S, Cannon JG, Ghorbani R, *et al. In vitro* production of IL-1β, IL-1α, TNF and IL-2 in healthy subjects: distribution, effect of cyclooxygenase inhibition and evidence of independent gene regulation. *Eur J Immunol* 1989; **19**: 2327–2333.
23. Rodorf-Adam C, Lazdins J, Woods-Cook K, *et al.* An assay for the detection of interleukin-1 synthesis inhibitors: effects of antirheumatic drugs. *Drugs Exptl Clin Res* 1990; **15**: 355–362.
24. Turner NC, Guermy T. The effect of phophodiesterase inhibitors on macrophage superoxide generation. *Thorax* 1991; **46**: P107.
25. Fuller RW, O'Malley G, Baker AJ, MacDermott J. Human alveolar macrophage activation: inhibition by forskolin but not β-adrenoceptor stimulation or phosphodiesterase inhibition. *Pulm Pharmacol* 1988; **1**: 101–106.
26. Komas N, Lugnier C, Andriantsitohaina R, Stoclet JC. Characterization of cyclic nucleotide phosphodiesterases from rat mesenteric artery. *Eur J Pharmacol* 1991; **208**: 85–87.
27. Brandwein SR. Regulation of interleukin 1 production by mouse peritoneal macrophages. *J Biol Chem* 1986; **261**: 8624–8632.
28. Torphy TJ, Livi GP, Balcarek JM, White JR, Chilton FH, Undem BJ. Therapeutic potential of isozyme-selective phosphodiesterase inhibitors in the treatment of asthma. *Advances in Second Messenger and Phosphoprotein Research* 1992; **25**: 289–305.
29. Broide DH, Lotz M, Cuomo AJ, Coburn DA, Federman EC, Wasserman SI. Cytokines in symptomatic asthma airways. *J Allergy Clin Immunol* 1992; **89**: 958–967.
30. Slungaard A, Vercellotte GM, Walker G, Nelson RD, Jacob HE. Tumor necrosis factor α/cachectin stimulates eosinophil oxidant production and toxicity towards human endothelium. *J Exp Med* 1990; **171**: 2025–2041.

ACKNOWLEDGEMENTS. We would like to thank Dr Caggianno for stimulating discussions, and critically reading the manuscript and Ms T. Coffey for statistical analysis. The secretarial expertise of Ms Jessica Noll is gratefully acknowledged.

**Received 8 September 1992; accepted in revised form 25 September 1992**
